# Supplementary material for: A slot region in the magnetosphere of Jupiter
Source: Nat Commun. 2025 Aug 25;16:7916. doi: 10.1038/s41467-025-63186-8 (PMC12379232; doi:10.1038/s41467-025-63186-8)
Supplement: Supplementary file 1 — Supplementary Information [file 41467_2025_63186_MOESM1_ESM.pdf]

Supplementary Materials for

A slot region in the magnetosphere of Jupiter

Authors:

Minyi Long[1,2], Elias Roussos[2*], Binbin Ni[1,3*], Qianli Ma[4,5*], Peter Kollmann[6], Ruoxian Zhou[7], George Clark[6], Norbert Krupp[2], Xing Cao[1], Peng Lu[1], Yixin Hao[2], Shaobei Wang[1]

Affiliations:

[1]School of Earth and Space Science and Technology, Wuhan University, Wuhan, China.

[2]Max Planck Institute for Solar System Research, Göttingen, Germany.

[3]CAS Center for Excellence in Comparative Planetology, Hefei, China.

[4]Center for Space Physics, Boston University, Boston, MA, USA.

[5]Department of Atmospheric and Oceanic Sciences, University of California, Los Angeles, CA, USA.

[6]Johns Hopkins University Applied Physics Laboratory, Laurel, MD, USA.

[7]Department of Physics, University of Texas at Dallas, Dallas, TX, USA.

[*] Correspondence to: roussos@mps.mpg.de; bbni@whu.edu.cn; qianlima@atmos.ucla.edu

This file includes:

Supplementary Text

Supplementary Figs. 1−5

Supplementary Table 1

This Supplementary Information accompanies the manuscript titled "A slot region in the magnetosphere of Jupiter". It provides additional analysis and figures supporting the main findings, including spatial correlation analysis, wave–particle interaction modeling, and refined coordinate descriptions.

Supplementary Text

Supplementary Fig. 1, we show the orbital configuration of Juno and Europa during the

observation interval shown in Figure 1. During this orbit, whistler-mode waves were observed from 20:00 to 22:30 UT, at magnetic latitudes ranging from ~20° to ~50°, with particularly intense amplitudes between 20:30 and 22:00 UT. Notably, Juno crossed the same magnetic field line at 21:37 UT that Europa had crossed earlier at 20:00 UT. This temporal and spatial proximity suggests that the observed wave enhancements are likely linked to localized coupling processes between Europa and Jupiter's magnetosphere, rather than indicating a broad, global effect. This point better aligns with our overall conclusion that while Europa can locally modify the surrounding plasma environment and wave activity, the global asymmetry patterns observed in Jupiter's magnetosphere appear largely unaffected by the moon's immediate influence.

As shown in Supplementary Fig. 2, the averaged wave amplitudes exhibit a sharp increase around Europa's orbit (M-shell = 8 - 10), corresponding to a decrease in omni-directional electron fluxes in the same region. The wave amplitudes are stronger on the dawnside compared to the duskside, consistent with the more pronounced depletion of electron fluxes in the dawn sector. The observed dawn-dusk asymmetries in both electron fluxes and whistler-mode waves are likely influenced by the large-scale dawn-dusk electric field[1–5]. This electric field drives inward transport of electrons to lower M-shells on the dayside and outward transport on the nightside. Electrons accelerated by inward transport on the dayside drift in Jupiter's rotational direction, leading to increased electron fluxes in the dusk sector and a corresponding decrease in the dawn sector. Meanwhile, the combination of the large-scale dawn-dusk electric field with the globally asymmetric distribution of whistler-mode wave amplitudes can further facilitate the observed profile of the dawn-dusk asymmetry in electron fluxes, which however requires four-dimensional (4-D) Fokker–Planck diffusion simulations for further exploration. Additionally, the averaged wave amplitudes near Io's orbit display a pronounced peak on the duskside, though observations in the dawn sector remain insufficient. A radially narrow decrease in energetic electrons, within a broader decrease, is observed along Io's orbit on the wake side of Io, which is consistent with the occurrence of whistler-mode waves[6]. While our primary focus is on the electron slot near Europa, we suggest that variations of energetic electron fluxes near Io's orbit likely follow similar mechanisms.

The traditional M-shell, as used in many previous studies, represents only the instantaneous traced

magnetic equator distance of Juno (or a moon) but is not a true invariant of drift motion. Using the M-shell to compare observations at different longitudes or local times may introduce systematic errors, as the same M-shell at different longitudes around Jupiter may not correspond to the same drift shell. Hence, in our investigation of local time asymmetries, we propose a new, simplified, and practical definition of the M-shell, based on the approximation that particle drifts occur at a nearly constant equatorial magnetic field strength. Specifically, the equivalent M-shell (M_{eq}) is defined as $(B_s/B_{eq})^{1/3}$, by assuming a surface magnetic field (B_s) of 4.28×10^5 nT.

We use the M_{eq} to investigate the whistler-mode wave and electron flux distributions in Supplementary Fig. 3. Overall, the statistical results are similar to those in Figs. 1. The intensification of wave amplitudes corresponds to a decrease in electron fluxes. However, the spatial coverage of wave amplitude enhancements extends to a broader range of M_{eq}, and the averaged wave amplitudes are more intense. This suggests that the effect of wave scattering may be stronger when considering the wave intensity on the M_{eq} grid.

Furthermore, we show in Supplementary Fig. 4 the spatial correlation between whistler-mode waves and energetic electron distributions in the (M_{eq}, MLAT)-space. There is a strong negative correlation between the averaged wave amplitudes and the latitudinally averaged f_N. The corresponding correlation coefficients (CC) are -0.92, -0.85, and -0.62 for each energy channel within $M_{eq} = 7.25 - 13.75$, with very low p-values (3.7×10^{-6}, 1.2×10^{-4}, and 1.7×10^{-2}). When using the parameter M_{eq} to analyze the spatial correlation, while the peak amplitudes and normalized electron fluxes shift in the coordinate position, the correlations remain consistent with the results shown in Fig. 2. These analyses indicate that the systematic differences introduced by use of M_{eq} are likely small for the investigation in this study. Given that the Juno community commonly uses the M-shell (M) as the mapping reference, we prefer to use M-shell rather than M_{eq} so that our results can be suitably mapped for comparisons with other published studies using Juno data. It is noted that M_{eq} can be considered as an improved mapping approximation for future researches.

We show the resultant bounce-averaged diffusion rates in Supplementary Fig. 5 by using the wave normal angle model shown in Supplementary Table 1. The bounce-averaged pitch angle diffusion rates are 1 - 2 orders of magnitude larger than the mixed and momentum diffusion rates. LFWs can pitch

angle scatter energetic electrons with energies greater than 100 keV through cyclotron resonance, and scatter electrons with energies around 10 keV to 1 MeV at large equatorial pitch angles (80° - 90°) via Landau resonance. HFWs can resonate efficiently with electrons of $\alpha_{eq} < 80°$ in the energy range from 10 keV to 100's keV. The combined effects of the two frequency bands suggest that whistler-mode waves act as the primary driver of electron loss in Jupiter's radiation belt near Europa's orbit.

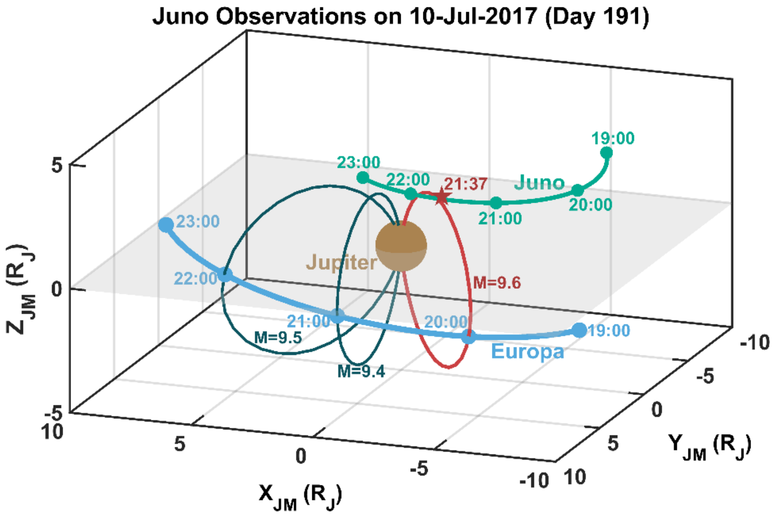

Supplementary Figure 1 | Three-dimensional trajectories of the Juno spacecraft and the Europa moon in Jupiter-centered magnetic (JM) coordinates during the interval of 10 July 2017 (Day 191). The sphere denotes Jupiter, and the field line illustrates a representative magnetic field line connected to Europa's orbit. Time labels along the trajectories mark universal time (UT) in hours, and M means M-shell (the distance from the magnetic equator to Jupiter's center, normalized by Jupiter's radius). The five-pointed star symbol indicates a key moment when Juno crossed a magnetic field line (UT=21:37) swept by Europa's orbital motion, occurring approximately 1.5 hours after Europa's closest magnetic (UT=20:00) mapping to Juno's position. Source data are provided as a Source Data file.

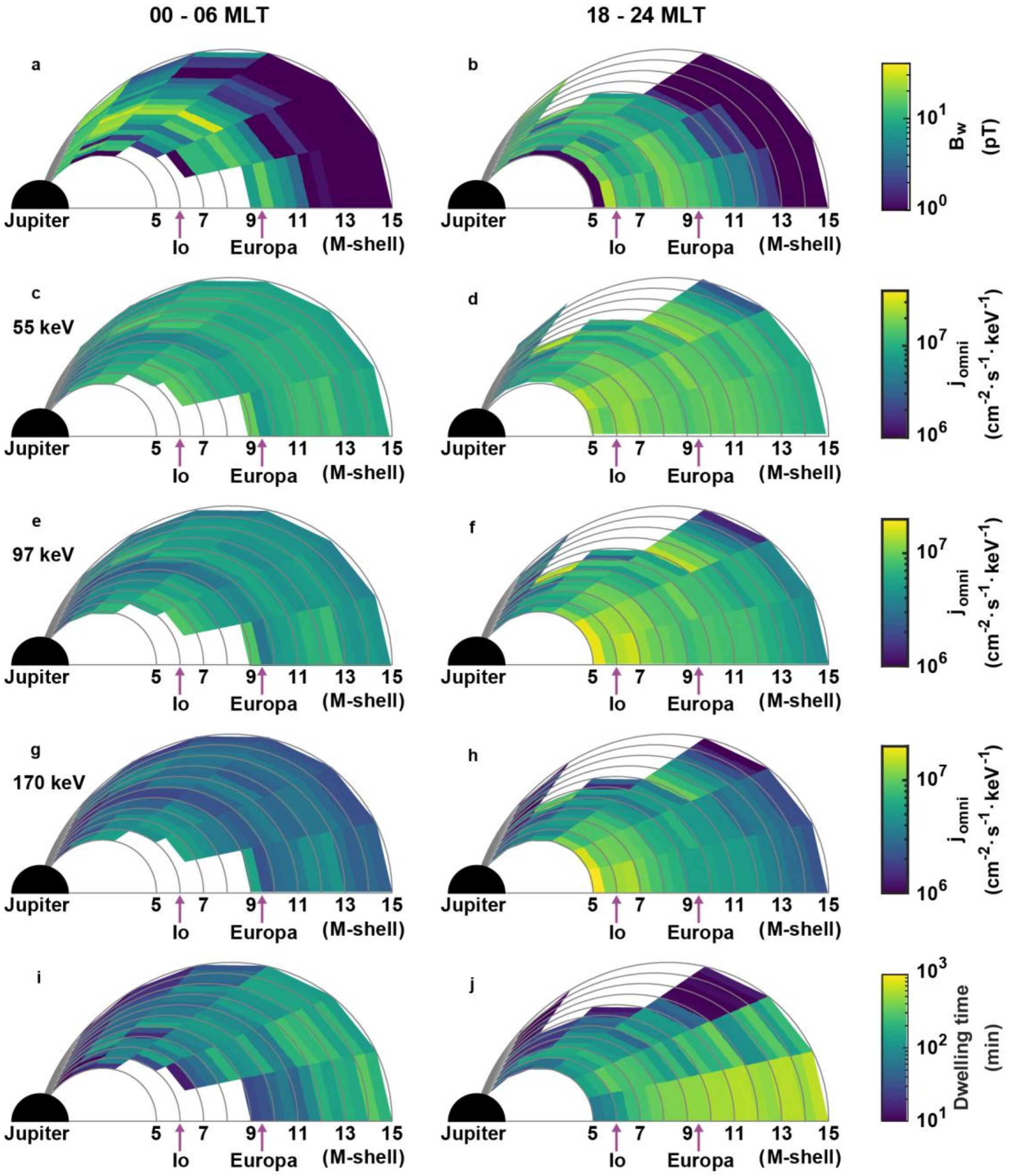

Supplementary Figure 2 | Statistics of whistler-mode waves and corresponding energetic electron fluxes as functions of M-shell (the distance from the magnetic equator to Jupiter's center, normalized by Jupiter's radius) and MLAT (magnetic latitude) in the magnetic meridian plane for different MLT (magnetic local time) sectors in Jupiter's magnetosphere. **a-b**, average wave amplitude distributions on the dawnside (00-06 MLT) and on the duskside (18-24 MLT). **c-h**, omni-directional electron flux distributions for three indicated energy channels (55, 97, 170 keV) on the dawnside and on the duskside. **i-j**, the corresponding spacecraft dwelling time. The magnetic latitude resolution is 10°, and the M-shell resolution is 0.5. Source data are provided as a Source Data file.

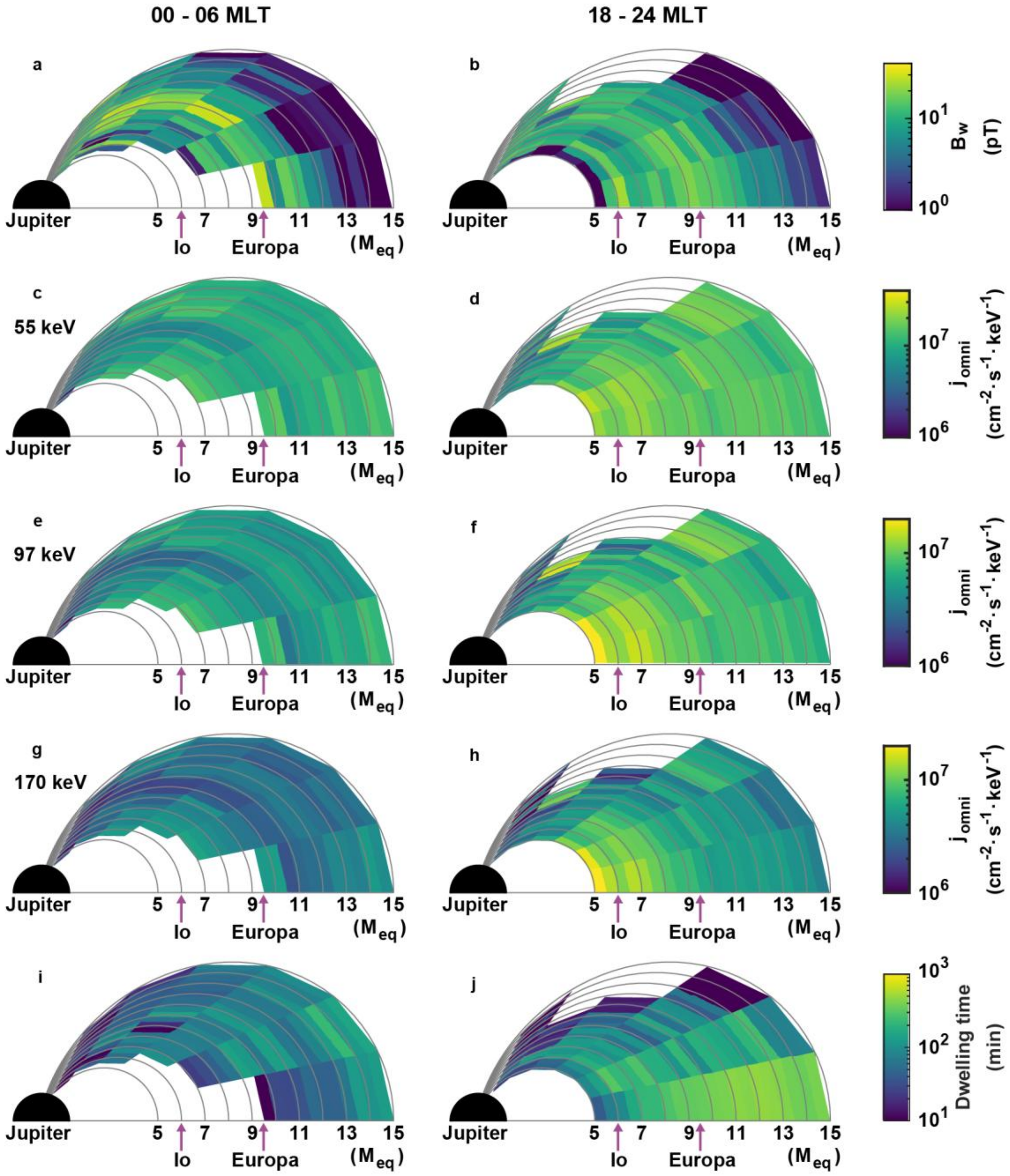

Supplementary Figure 3 | Statistics of whistler-mode waves and corresponding energetic electron fluxes as functions of M_{eq} (the cube root of the local-to-equatorial magnetic field strength ratio along the same field line) and MLAT (magnetic latitude) in the magnetic meridian plane for different MLT (magnetic local time) sectors in Jupiter's magnetosphere. **a-b**, average wave amplitude distributions on the dawnside (00-06 MLT) and on the duskside (18-24 MLT). **c-h**, omni-directional electron flux distributions for three indicated energy channels (55, 97, 170 keV) on the dawnside and on the duskside. **i-j**, the corresponding spacecraft dwelling time. The magnetic latitude resolution is 10°, and the M-shell resolution is 0.5. Source data are provided as a Source Data file.

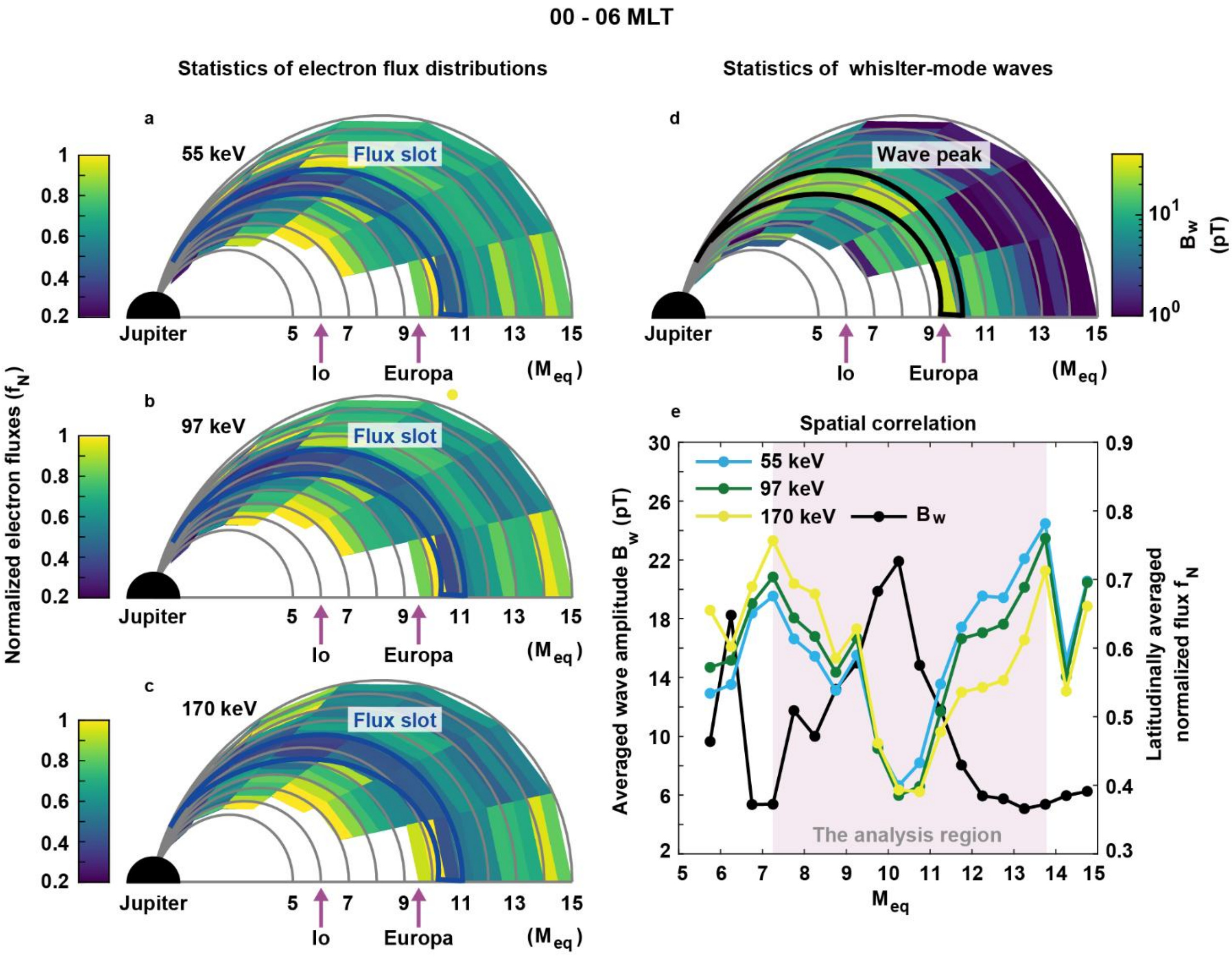

Supplementary Figure 4 | Spatial correlation of energetic electron fluxes and whistler-mode wave amplitudes (B_w) in the neighboring environment of Europa. MLAT denotes magnetic latitude, MLT means magnetic local time, and the M_{eq} represents the cube root of the local-to-equatorial magnetic field strength ratio along the same field line. **a-c**, the normalized electron fluxes in the magnetic meridian plane (Δ|MLAT| = 10°) for the three specified energy channels (55, 97, 170 keV) within the equivalent M-shell range of 5 − 15 (ΔM_{eq} = 0.5) in the dawn sector (MLT = 00 – 06). The normalized flux (f_N) is the ratio between the omni-directional electron fluxes (j_{omni}) and the maximum j_{omni} within each magnetic latitude bin within 5 < M_{eq} < 15. **d**, the averaged whistler-mode wave amplitudes in the magnetic meridian plane on the dawnside. Flux slot indicates the region of reduced electron fluxes, while wave peak highlights the region with enhanced wave amplitudes. **e**, spatial correlation between latitudinally averaged normalized electron fluxes for the three energy channels and averaged wave amplitudes, calculated within the shaded region of M_{eq} = 7.25 - 13.75 (shaded region). Due to limited data coverage within M-shell < 9 within |MLAT| < 10°, the latitudinally averaged normalized electron fluxes and wave amplitudes are calculated within |MLAT|= 10° − 60°. Source data are provided as a Source Data file.

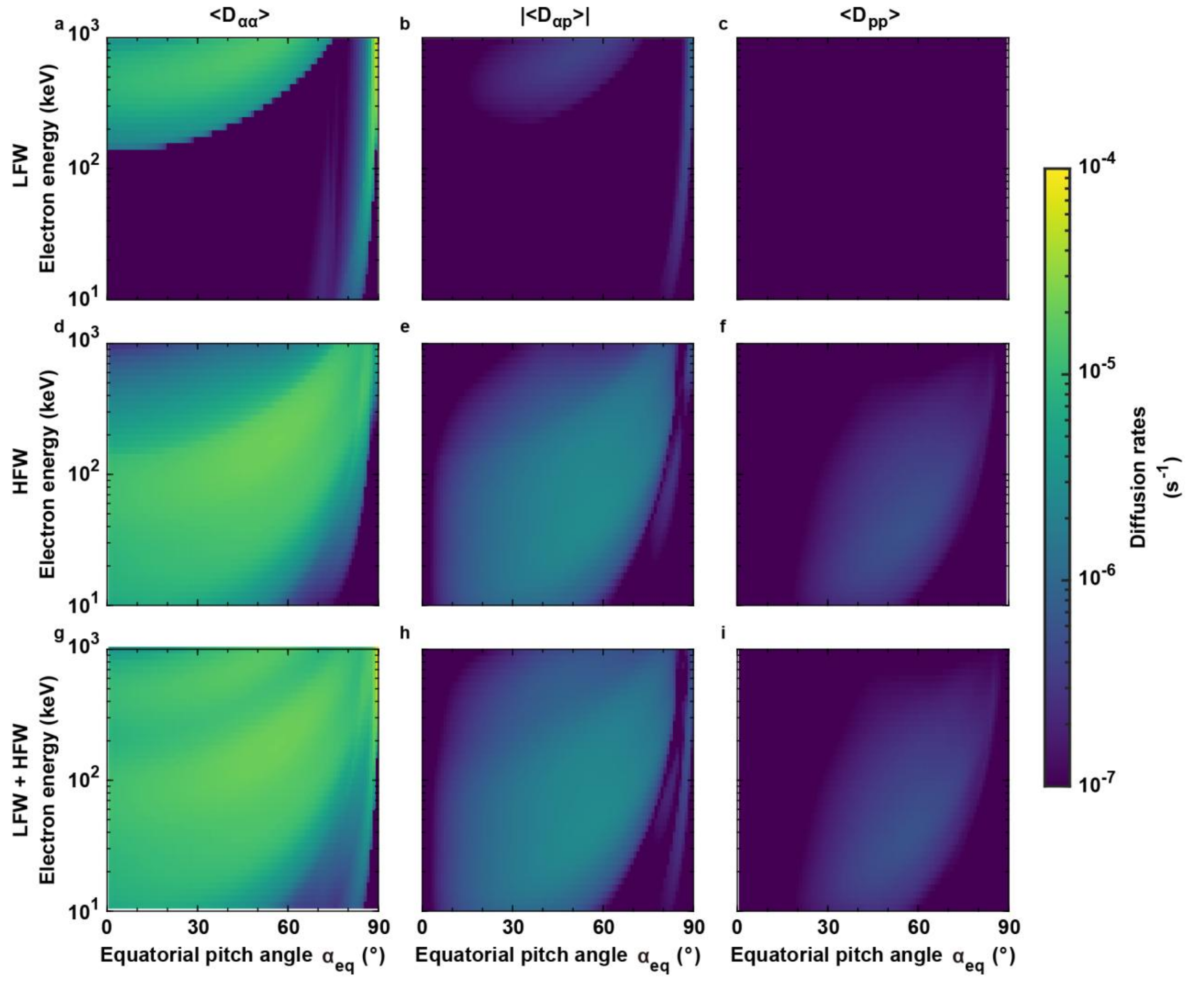

Supplementary Figure 5 | 2-D plots of bounce-averaged diffusion rates induced by whistler-mode waves at Jovian M-shell = 9.5 (M-shell is the distance from the magnetic equator to Jupiter's center, normalized by Jupiter's radius), as a function of electron kinetic energy and equatorial pitch angle. From left to right, the panels show bounce-averaged pitch angle diffusion rates ($<D_{\alpha\alpha}>$), cross diffusion rates ($<D_{\alpha p}>$), and momentum diffusion rates ($<D_{pp}>$). From top to bottom, the panels display the diffusion rates due to LFWs (low frequency whistler-mode waves), HFWs (high frequency whistler-mode waves), and their combined scattering coefficients. Source data are provided as a Source Data file.

Supplementary Table 1 | Wave normal angle models used for calculating diffusion rates induced by whistler-mode waves. MLAT is magnetic latitude, the LFWs are low frequency whistler-mode waves, the HFWs are high frequency whistler-mode waves. ψ_m denotes the maximum wave normal angle, and $\delta\psi$ represents the angular width. ψ_{lc} and ψ_{uc} indicate the lower and upper limits of the wave normal angle range, respectively. Source data are provided as a Source Data file.

Waves	MLAT (°)	ψ_m (°)	$\delta\psi$ (°)	ψ_{lc} (°)	ψ_{uc} (°)
LFWs	0-5	0	15	0	25
	5-10	20	15	0	40
	10-15	40	20	0	55
	15-20	50	30	15	70
	20-25	60	40	30	75
	25-30	70	50	50	80
	30-35	80	60	65	85
	35-40	80	70	75	85
	40-60	80	80	80	85
HFWs	0-20	0	10	0	30

References

1. Krupp, N. et al. Local time asymmetry of energetic ion anisotropies in the Jovian magnetosphere. Planet. Space. Sci. **49**, 283–289 (2001).
2. Roussos, E. et al. Drift-resonant, relativistic electron acceleration at the outer planets: Insights from the response of Saturn's radiation belts to magnetospheric storms. Icarus **305**, 160–173 (2018).
3. Hao, Y. et al. The formation of Saturn's and Jupiter's electron radiation belts by magnetospheric electric fields. Astrophys. J. **905**, L10 (2020).
4. Wang, J. et al. Dawn-dusk asymmetry of plasma flow in Jupiter's middle magnetosphere observed by Juno. Geophys. Res. Lett. **51**, e2024GL110209 (2024).
5. Yuan, C. et al. Galileo observation of electron spectra dawn-dusk asymmetry in the middle Jovian magnetosphere: Evidence for convection electric field. Geophys. Res. Lett. **51**, e2023GL105503 (2024).
6. Paranicas, C. et al. Io's effect on energetic charged particles as seen in Juno data. *Geophys. Res. Lett.* **46**, 13615–13620 (2019).
